# Volumetric Assessment of Resorption Patterns of Bilateral Alveolar Clefts in Cone‐Beam Computed Tomography in Two‐ Stage Bone Graft

**DOI:** 10.1111/ocr.12902

**Published:** 2025-02-04

**Authors:** Tobias Regnstrand, Alice Bousiou, Agneta Karsten, Daniel Benchimol, Reinhilde Jacobs

**Affiliations:** ^1^ Division of Oral Diagnostics and Surgery Unit, Department of Dental Medicine Karolinska Institutet Stockholm Sweden; ^2^ Division of Orthodontics, Department of Dental Medicine Karolinska Institutet Stockholm Sweden; ^3^ OMFS‐IMPATH Research Group, Department of Imaging and Pathology, Faculty of Medicine Catholic University of Leuven Leuven Belgium

## Abstract

**Objective:**

Few studies have analysed the outcome of bone grafts in bilateral alveolar clefts and the bone fill with a two‐step surgery method. The currently applied three‐dimensional method used in this study enables a comprehensive description of the bone fill of bilateral clefts after bone grafting. The study aimed to describe alveolar cleft volume and bone fill after alveolar bone grafting of bilateral alveolar clefts treated with two‐step bone grafting, with a comparison between the first and the second bone graft site. A secondary aim was to investigate whether the cleft volume on the non‐surgical side changed after contralateral surgery.

**Materials and Methods:**

In this retrospective study, 60 CBCT scans from 20 patients were included (8 girls and 12 boys) with an age range of 6.5–11.5 years (mean age 8.7). The cleft volume was measured in pre‐ and post‐operative CBCT scans and assessed in ITK‐SNAP to calculate the bone fill of the cleft.

**Results:**

After bone grafting, 47% of the first bone‐grafted cleft was filled with bone, and 33% of the second bone‐grafted cleft, without significant difference between them (*p* = 0.03). The mean preoperative cleft volume was 0.42cm^3^ and the mean residual cleft volume after bone graft was 0.23cm^3^. There was however a significant difference when comparing the bone fill between the nasal and the dental part (*p* < 0.001).

**Conclusion:**

Almost half of the cleft volume was filled with bone after bone grafting. The order of the bone graft side did not influence the bone fill of the cleft.

## 1 Introduction

Worldwide cleft lip and palate (CLP) affects one in 700 children [1].

Around 75% of patients born with CLP have an alveolar cleft defect [2]. The alveolar cleft defect is a result of incomplete fusion of the medial frontonasal process with the lateral maxillary process. Approximately 80% of patients with CLP are unilateral and 20% are bilateral [3].

Bone graft of the alveolar cleft is usually performed in the mixed dentition stage, before the eruption of the permanent lateral or canine between 8 and 12 years of age [4]. Dental anomalies and agenesis, especially upper laterals, are common among children born with alveolar clefts [5]. The lateral adjacent to the cleft is missing in 44% among children with unilateral cleft [5]. Ideally, secondary alveolar bone grafting is performed after the eruption of the lateral incisor but before the eruption of the canine, so that the canine can erupt through the graft [6]. The cleft side with the canine closest to the cleft or the larger cleft is bone grafted first and the next graft procedure is performed at least 6 months after the initial procedure [7]. The primary goal of the treatment is to provide bone for tooth eruption, close oronasal fistulas and to unify and stabilise the maxilla [8].

Sufficient diagnostic imaging of patients with alveolar clefts is valuable for estimation of the size of the alveolar cleft defect and facilitating the treatment [9, 10]. Before bone grafting (pre‐surgery), diagnostic imaging facilitates evaluation of the size of the alveolar cleft, the position of adjacent teeth and their development and eruption pattern [10]. After bone grafting (post‐surgery), diagnostic imaging facilitates the evaluation of the alveolar bone, the eruption of the lateral incisor or canine next to the cleft [9].

Intraoral imaging has been widely used pre‐ and post‐surgery to estimate cleft size and post‐surgery to evaluate the resulting alveolar bone graft [9, 11]. Yet, a limitation of two‐dimensional (2D) radiography is that it is not possible to assess the cleft in bucco‐palatal direction, making it difficult to accurately evaluate the cleft area three‐dimensionally [12]. This could be overcome by three‐dimensional (3D) imaging. However, only few studies on 3D assessment of bilateral cleft lip and palate (BCLP) [13, 14], probably because of the lower prevalence of BCLP compared to unilateral cleft lip and palate (UCLP).

The significance of this study was to three‐dimensionally assess the pre‐surgical site in patients with bilateral cleft and to study the differential outcome between the first and second bone grafting sites. Also, to clarify if the cleft volume on the non‐surgical side (before intervention on this side) changed after surgery on the contralateral side.

## 2 Material and Methods

### 2.1 Study Design

For this retrospective cohort study, CBCT scans taken pre‐ and post‐operative bone graft on 20 children with bilateral clefts with alveolar defects undergoing alveolar bone graft surgery were included. No further subdivision of the extent of the cleft was performed. The CBCT scans were retrospectively collected from Romexis (Planmeca Oy, Finland, Helsinki). The CBCT examinations were performed between 2016 and 2021 at the Clinic of Maxillofacial Radiology at Karolinska Institutet after referrals from the Craniofacial Centre at Karolinska University Hospital, where the bone grafting was performed. The Craniofacial Centre at Karolinska University Hospital used a two‐stage method for bone grafting bilateral clefts, one cleft was bone grafted at a time. All bone grafting procedures were performed by plastic surgeons with the iliac crest as donor site for the bone graft.

### 2.2 Inclusion and Exclusion criteria's

The inclusion criteria for this study were:

- Three CBCT scans per subject: pre‐alveolar graft surgery, post‐first alveolar graft surgery, and post‐second alveolar graft surgery.
- Scans with full depiction of the cleft area, adjacent teeth, nasal floor, spina nasalis anterior as well as completely visible and unerupted maxillary canines.

CBCT scans were excluded if:

- Scans confirmed erupted canines.
- Presented poor quality, for example, un‐sharp images due to movement during exposure that inhibited the accuracy of measurements.

All CBCT examinations were performed using a Planmeca Promax 3D MID unit (Planmeca Oy, Helsinki, Finland) operating at 90 kV (tube voltage) and 4.5–8 mA (tube current) with an exposure time of 4–12 s depending on which protocol that was used. The volumes or field of view (FOV) were 8 × 5 cm with a voxel size of 200μm [15].

CBCT scans were analysed independently by two observers, a specialist in dental maxillofacial radiology and a general dentist working at the Clinic of Oral Radiology at Karolinska Institutet, Huddinge, Sweden.

### 2.3 Study Parameters

**FIGURE 1:**
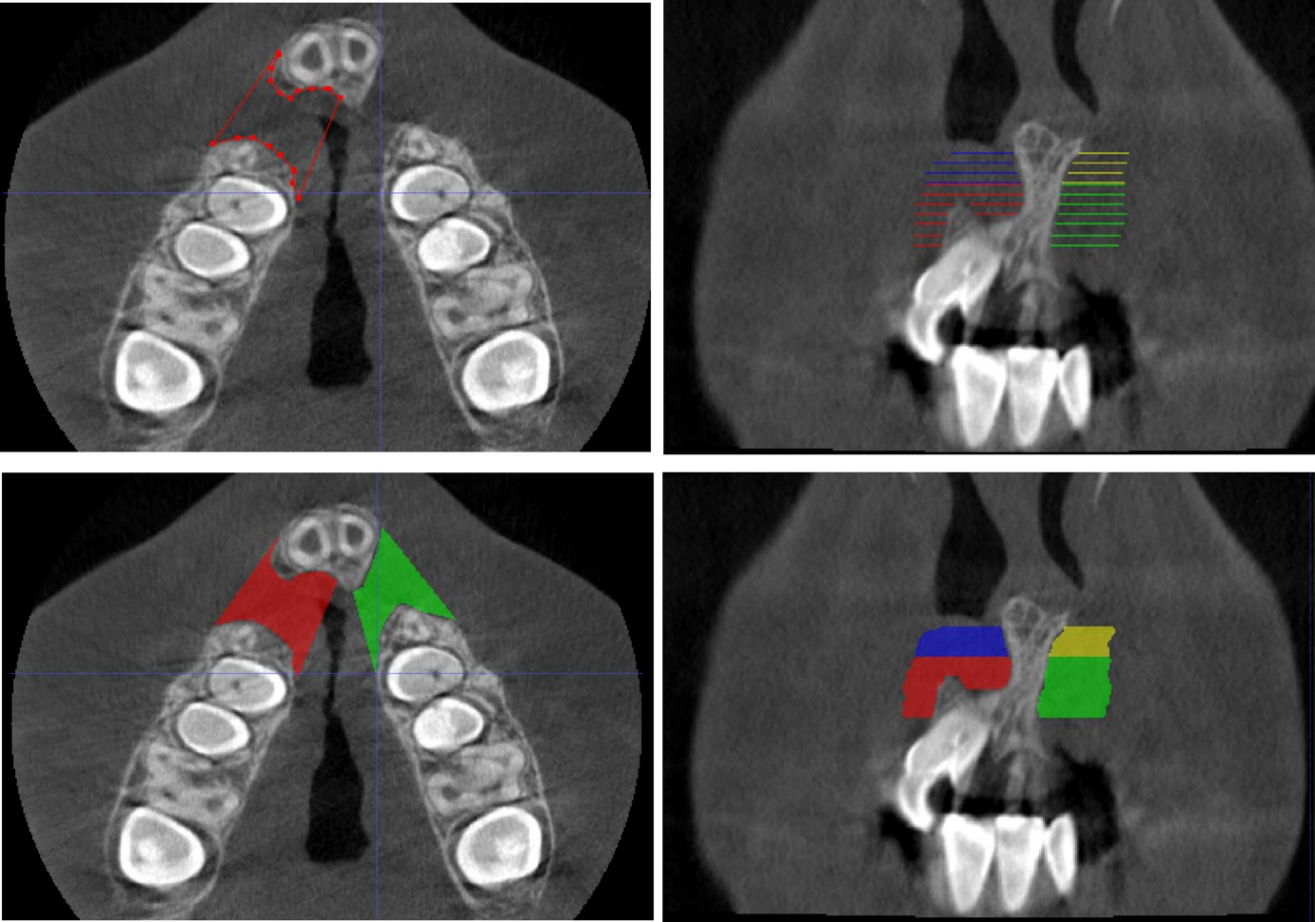
Manual segmentation of the cleft area at different levels performed every mm in Cone‐beam Computed Tomography scan using the software ITK‐SNAP (upper images). Control of the accuracy of the segmented cleft volumes after interpolation of the superior and inferior volumes in Cone‐beam computed tomography scan using the software ITK‐SNAP (lower images). The blue and yellow areas are the superior part of the cleft, while the red and green are the inferior part.

Segmentation was performed in the axial plane. Boundaries were defined as follows:

- Superior boundaries: 1 mm inferior of spina nasalis anterior.
- Inferior boundaries: cementoenamel junction of the adjacent medial incisor.
- Lateral boundaries: straight line between the anterior and posterior alveolar bone segment.
- Medial boundaries: straight line between the posterior alveolar bone segment and the posterior border of the incisive foramina.
- The segmentation of the cleft volume was divided into one superior part above the apex of the adjacent central incisor, and one inferior part (below the apex of the central adjacent incisor, see Figure 1).

The CBCT scans were exported as DICOM stacks from Romexis 4.6.0.R (Planmeca Oy, Helsinki, Finland) and was imported to the software ITK‐SNAP version 3.8.0. The alveolar cleft was segmented by using the ‘polygon mode’ in the axial view each millimetre, the segmentations were then interpolated to a volume with the ‘volumes and statistics’ tool. After interpolation, a manual control was performed on the accuracy of the interpolation of the cleft borders (Figure 1).

The anterior border of the cleft was defined by manually drawing a straight line between the buccal aspect of the lateral segment to the buccal aspect of the medial segment. The posterior border was defined by manually drawing a straight line from the anterior aspect of the incisive foramina to the palatal aspect of the lateral segment. The bone fill is calculated by dividing the postoperative volume with the preoperative volume, this number is then subtracted from one to get the bone fill. The study was ethically approved by the Swedish Ethical Review Authority (Dnr 2021‐04521) and complies with the Declaration of Helsinki.

### 2.4 Statistical Analysis

For statistical analyses, a paired *t*‐test was used for comparisons between cleft volumes. A one‐way analysis of variance (ANOVA) test was applied to detect statistically significant differences between different areas within the cleft volumes. Prior to statistical analysis of bone fill, the data was log‐transformed, and Bonferroni correction was applied, with significant differences detected for *p*‐values below 0.01. Inter‐ and intra‐observer reliability was assessed using the Intra‐class correlation coefficient (ICC). For statistical analyses, IBM SPSS Statistics for Windows, Version 27 (Armonk, NY) was used.

## 3 Results

**FIGURE 2:**
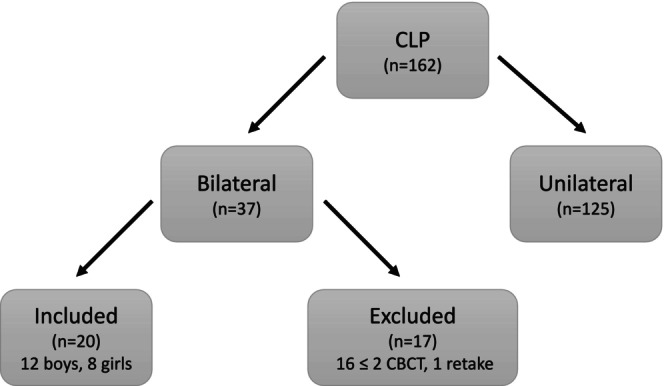
Flowchart over included and excluded patients.

Between 2016 and 2021, a total of 162 patients born with CLP were radiologically examined at the Department of Dental Medicine, Karolinska Institutet, Sweden with 37 being patients with BCLP. Of these, a total of 20 patients with bilateral cleft with three CBCT each, 8 girls and 12 boys were included in this study (Figure 2). The mean age at the time of the first CBCT was 8.7 years for boys (age range 6.7–11.5 years), and 8.9 years for girls (age range 7.6–9.9 years). The mean time between the 1st and 2nd CBCT was 12.5 month and 13.9 month between the 2nd and 3rd CBCT. Bone‐grafting procedures were performed by four different plastic surgeons. Aplasia of both lateral incisors were seen in 25 clefts (12 clefts on the right side and 13 clefts on the left side). The lateral incisors were located laterally in 12 clefts (six clefts on the right side and five clefts on the left side). In three clefts, lateral incisors were seen both lateral and medial of the cleft (one cleft on the right side and three clefts on the left side).

**FIGURE 3:**
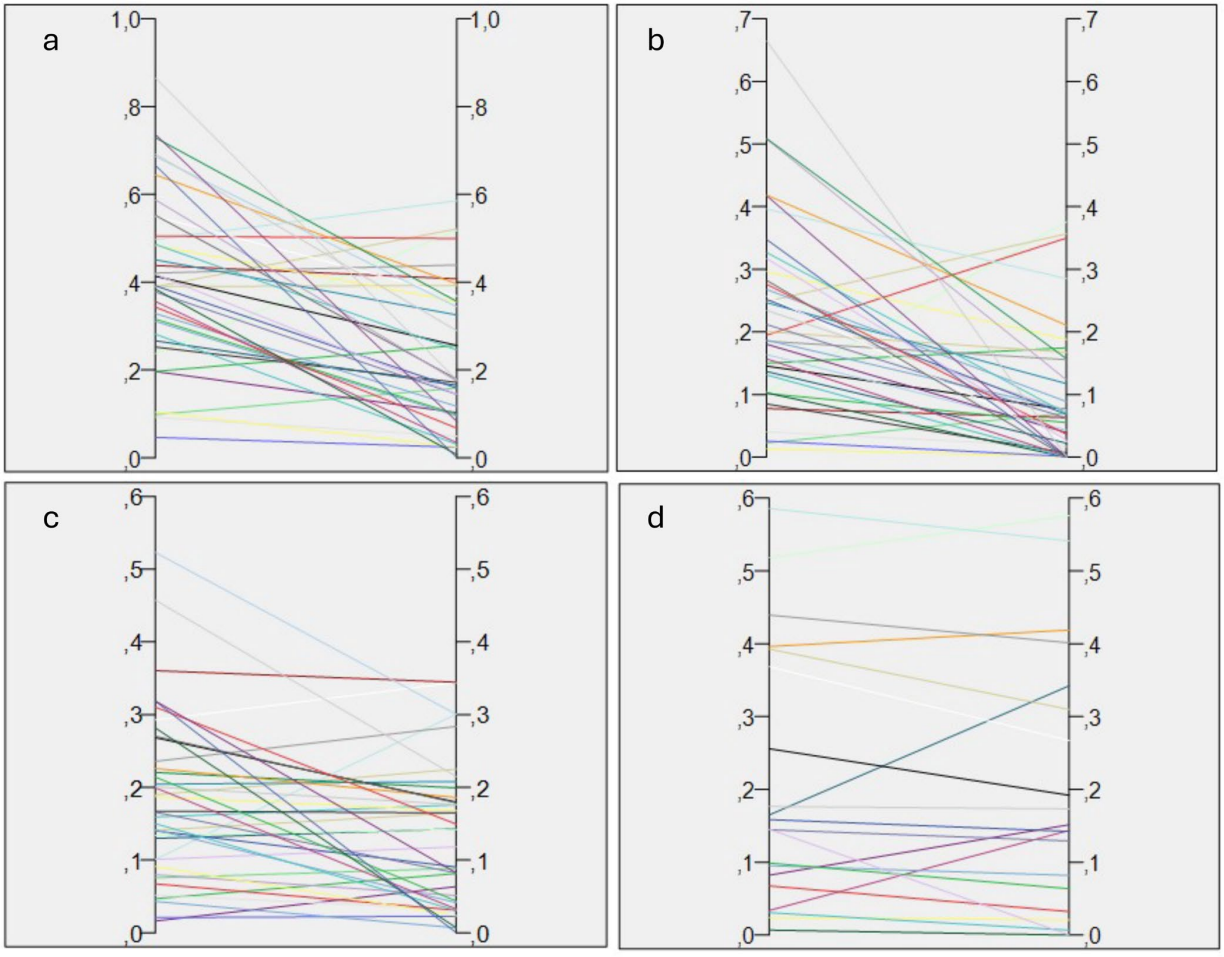
Volumetric change (cm^3^), preoperative on the left side, and postoperative on the right side. One line for each cleft. (a) total pre‐ and postoperative cleft volume, (b) dental volume pre‐ and postoperative, (c) nasal volume pre and postoperative, (d) cleft volume of the first cleft between the postoperative CBCT and 3rd CBCT.

The mean residual cleft volume of the first operated alveolar cleft was 0.2 cm^3^ and 0.24 cm^3^ for the second operated cleft with no significant difference between them (*p* = 0.03). The mean preoperative volume was 0.42cm^3^, and 0.23cm^3^ postoperative bone graft (Tables 1 and 2). The cleft volume in the passive cleft did not change after surgery on the contralateral side (*p* > 0.05) with a mean volume of 0.41 cm^3^ preoperatively and 0.42 cm^3^ postoperatively (Figure 3). One case had atypical anatomy which made it impossible to analyse the dental and nasal volume separately, only total cleft volume was registered in this case. The overall mean bone fill was 45% for both clefts together. For calculation of the bone fill for the inferior and superior segment, two outliers were excluded for the inferior part, and two outliers were excluded from the superior part. Our definition of an outlier was if the postoperative volume was more than double the preoperative volume. For the volumetric comparisons, all data was included in the analysis. The bone fill of the inferior part of the cleft was 62% (52% with outliers included) with a mean preoperative inferior volume of 0.23cm^3^, and 0.1cm^3^ postoperative. For the superior part of the cleft, the bone fill was 29% (12% with outliers included) with a preoperative superior volume of 0.19cm^3^ and 0.13cm^3^ postoperative (Table 2). No statistically significant difference was seen between the pre‐ or postoperative volume between the first and second cleft (*p* > 0.05). However, there was a significant difference when comparing the bone fill between the superior and the inferior part (*p* < 0.001). When comparing the cleft volume of the first alveolar cleft between the 2nd and 3rd CBCT, no significant difference in cleft volume were seen (*p* = 0.5).

**TABLE 1:** Mean cleft volume (cm^3^) prior to and post‐bone graft.

	Superior volume	Inferior volume	Total volume	*N*
1st preoperative volume				
Mean	0.18	0.24	0.42	20
Std. Deviation	0.08	0.15	0.18	
Passive preoperative volume				
Mean	0.19	0.22	0.41	20
Std. Deviation	0.11	0.16	0.22	
1st Postoperative volume				
Mean	0.13	0.09	0.21	20
Std. Deviation	0.10	0.11	0.18	
2nd preoperative volume				
Mean	0.19	0.22	0.42	20
Std. Deviation	0.14	0.14	0.21	
Passive postoperative volume				
Mean	0.19	0.22	0.42	20
Std. Deviation	0.14	0.14	0.21	
2nd postoperative volume				
Mean	0.14	0.11	0.25	19
Std. Deviation	0.09	0.13	0.17	
Total preoperative volume				
Mean	0.19	0.23	0.42	40
Std. Deviation	0.11	0.14	0.19	
Total postoperative volume				
Mean	0.13	0.09	0.23	39
Std. Deviation	0.1	0.10	0.16	

**TABLE 2:** Descriptive statistics of bone fill post‐bone graft. The corrected data exclude clefts or part of clefts with a postoperative volume more than twice as large as the preoperative volume.

	Superior	Inferior	Total	*N*
1st cleft	24%	55%	47%	20
Corrected	37%	72%	54%	18
2nd cleft	2%	38%	33%	19
Corrected	18%	51%	35%	17
Total	12%	52%	40%	39
Corrected	29%	62%	45%	35

The inter‐observer agreement was 0.91 (ICC) while the intra‐observer agreement was 0.93 (ICC) for Observer 1 and 0.89 (ICC) for Observer 2.

## 4 Discussion

The result of the present study indicated that post‐operative alveolar cleft volume does not differ between the first and the second graft site in patients with bilateral cleft. There was however significantly less bone fill in the superior part of the cleft as compared to the fill in the inferior part (*p* < 0.01). The present study also concluded that two‐step bone grafting procedures did not affect the cleft volume on the passive side.

Previous studies have assessed bone fill in unilateral clefts, with bone fill of the cleft after 1 year varying between 36%–99% [16, 17, 18, 19]. Studies assessing bone fill in bilateral clefts are far less common [13, 14]. One study assessing alveolar clefts treated with bone grafts from iliac crest, found that bone fill after 1 year was approximately 45% for bilateral clefts and 70% for unilateral clefts [20]. This corresponds well with the results of the present study. In this context, follow‐up time for the postoperative CBCT examination may have an influence on the bone fill. The largest resorption of the bone graft occurs during the first year with a stabilisation of the bone graft the following 2 years [21]. With unerupted canines, there was 56% resorption after 2–3 years while 44% resorption occurred if the canine had erupted [22]. The bone fill of the superior part of the cleft was lower in than in the inferior part, this result in line with a previous study of unilateral clefts with > 75% bone fill in the dental part and < 50% in the nasal part [23].

While bone fill of the cleft gives an indication of the outcome of the surgery, several other factors influence the outcome of orthodontic treatment and the occlusion. A high percentage of bone fill is not automatically regarded as success as the term success itself is debated. Some authors considered it a success if oronasal fistulae disappeared even if the graft almost was not present anymore, whereas others considered this as a failure [20]. The decision if the cleft needs to regraft is dependent on what is planned for the cleft area, for example if a tooth is planned to be orthodontically moved into the cleft area. To decide which cleft to bone graft first, this decision was taken depending on if the lateral or the canine was supposed to erupt into the alveolar cleft and which cleft area that had the crown of the lateral tooth closest to the cleft. If it was similar on both sides, the largest cleft was bone grafted first. For the patients in the present study, decision for potential regraft were taken on multidisciplinary rounds. Often, the marginal bone level of the teeth adjacent to the cleft is used as a reference for deciding about success or failure of alveolar bone grafting [18]. Also, canine eruption was found to be correlated with success [12].

Measuring the cleft volume in bilateral clefts can be challenging because of variance in morphology and how to define the borders of the clefts. In addition, bilateral cases have no unaffected contralateral side to use as a reference. According to Stoop et al. the palatal boundary of the alveolar cleft defect is especially difficult to establish [1]. Despite this, the observers in the present study showed excellent inter‐ and intra‐observer agreement. There is no consensus in which anatomical landmarks to use for defining the cleft, this makes it challenging to compare our results to other studies. Good agreement were seen in a previous study comparing volumetric bone‐fill of the cleft and the Soumalainen index [24, 25].

When evaluating secondary alveolar bone grafting outcomes, 3D radiographic methods are more precise and reliable than 2D radiographs, especially for assessing bucco‐palatal bone status [12, 17, 26]. According to a systematic review by Yu et al., the outcome in intra oral images was overestimated in comparison to tomographic imaging, especially the bucco‐palatal width [12]. CBCT revealed that bucco‐palatal resorption was more frequent than vertical bone resorption, indicating that bone fill is overestimated when only the height of interalveolar bone is measured [12]. Agreement regarding the outcome of bone grafts between CBCT and intraoral images were seen in 41% of the cases, while intraoral images overestimated the bone in 51% of the cases [26]. Today, the advantage of 3D imaging is clear, as it is the only technique that depicts the cleft area in 3D, thus enabling a more thorough evaluation of the cleft.

However, one must consider that the use of 3D radiography leads to higher radiation doses in comparison to 2D radiography. According to Jacobs et al. patients with CLP have a three to five times higher cumulative radiation dose from dental radiology compared to age‐matched healthy controls [27]. When using CBCT or CT for 3D assessment of alveolar cleft defects, the smallest volume size compatible should be selected for the reduction of radiation dose. The surgery of the bilateral clefts assessed in the present study was performed with a two‐stage method which gives three CBCT for pre‐ and postoperative assessment. For dose reduction, bone grafting of both clefts at the same time would reduce the dose by 1/3 because only two CBCT examinations for pre‐ and post‐operative assessment would have been needed. Furthermore, a two‐stage method (in comparison to a one stage method) leads to additional anaesthetic, surgery, hospitalisation, and health care costs.

A limitation of the present study is that the bone grafting procedures in this study were performed by four experienced plastic surgeons. The possible difference in outcome depending on the operator was not investigated. A challenge with volumetric evaluation is the inability to differentiate between change of cleft volume of bone grafting from other potential factors such as to orthodontic interventions and natural growth. Although, the Craniofacial Centre at Karolinska University Hospital only performs minor orthodontic movement (if necessary) in other regions of the mouth so as not to disturb the healing of the bone graft until the postoperative CBCT examination. Therefore, orthodontic treatment performed between the CBCT examinations are not taken into consideration in the present study. Another limitation, due to the content of the ethical approval, was that no further subdivision of the extent of the clefts was performed.

A rather surprising finding was the increase of the cleft volume found in four cases that had postoperative cleft volumes more than twice as large as the preoperative volume. These were therefore excluded in the corrected bone fill calculation. Possible explanations for this may be the growth in combination with surgery if the premaxilla has been moved to a more favourable location.

In future studies, AI or automatic border recognition may be used for the superimposition of the volumes for a more thorough assessment of the volumetric change. However, this can be challenging due to growth and the bone graft itself. Therefore, superimposition was not used in the present project. In addition, in future studies, a larger cohort is desirable with further attention to patient characteristics and ethnicity. It would also be valuable to have a control group of patients with unilateral clefts to compare with, even though one must take into consideration that only 20% of orofacial clefts are bilateral. Finally, surgical variability and various surgical and orthodontic treatment approaches might be studied in the light of the desired outcome for the bone graft and the achieved orthodontic treatment result.

## 5 Conclusions

Almost 50% of the cleft volume in bilateral alveolar clefts is filled with bone after secondary alveolar bone grafting 1 year post surgery, with the largest amount of bone fill in the inferior part of the cleft. The order of the bone graft does not influence the bone fill of the clefts, suggesting that the preoperative cleft size may not need to be considered when deciding which cleft to operate first.

## Conflicts of Interest

The authors declare no conflicts of interest.

## Data Availability

The data that support the findings of this study are available on request from the corresponding author. The data are not publicly available due to privacy or ethical restrictions.
